# Directivity Maximization of Difference Patterns for Monopulse Microstrip Patch Arrays with Sidelobe Constraints

**DOI:** 10.3390/mi17030321

**Published:** 2026-03-04

**Authors:** Weizong Li, Yong-Chang Jiao, Yixuan Zhang, Li Zhang

**Affiliations:** National Key Laboratory of Radar Detection and Sensing, Xidian University, Xi’an 710071, China; wzli_1@stu.xidian.edu.cn (W.L.); yxuzhang@foxmail.com (Y.Z.); lizhang@mail.xidian.edu.cn (L.Z.)

**Keywords:** difference pattern (DP), directivity maximization, monopulse array, microstrip patch antenna, radome-enclosed linear phased array, planar phased array, low sidelobes

## Abstract

High-performance difference patterns (DPs) are critical for compact and integrated microwave array systems, particularly in monopulse tracking and beam-scanning applications. However, the design of monopulse phased arrays with steep slopes, high directivity, low sidelobes, and symmetric main lobes remains challenging due to constraints imposed by the array aperture and radome structure. In this paper, a novel design method is proposed to maximize the DP directivities for monopulse linear and planar phased arrays composed of microstrip patch antennas. The DP synthesis problem is first formulated as a nonconvex optimization model for directivity maximization. By fixing the reference phase of the DP slope and applying a first-order Taylor expansion of the quadratic function, the original problem is decomposed into a sequence of convex subproblems that can be solved efficiently. The proposed method fully exploits the flexibility of the phased array feed network, enabling directivity enhancement without altering the geometric configuration of the monopulse array. Finally, three numerical examples employing a radome-enclosed linear array, a uniform planar array, and a radome-enclosed planar array are presented to demonstrate the effectiveness of the proposed method in achieving the monopulse array DP synthesis with high directivity and symmetric main lobes.

## 1. Introduction

Antenna arrays with sum and difference patterns (SDPs) are widely employed in various radar and guidance systems for target detection and tracking [1,2]. In a typical monopulse architecture, the target angular and distance information is obtained by comparing the SDP signals. A difference pattern (DP) is applied to detect the direction of the target. Consequently, a low sidelobe level (SLL) DP with a large slope in the target direction is desired to enhance the tracking accuracy.

Many efficient DP synthesis techniques have been proposed, including numerical analysis methods, global optimization algorithms, convex optimization methods, and alternating projection techniques. Classical Bayliss method and its extensions were proposed in [3,4,5,6] to synthesize low SLL DPs for linear and planar arrays. Although these methods are computationally efficient and straightforward to implement, they are only applicable to uniform arrays composed of ideal isotropic point sources. With the development of computer technology, different kinds of global optimization algorithms [7,8,9,10,11,12,13], such as genetic algorithms [7,8], differential evolution algorithms [9,10], and particle swarm optimization methods [11], have been extensively applied to solve the DP synthesis problems. These global optimization methods are not limited by the array geometry and can obtain good results, but these algorithms may trap into local optimal solution, and the optimization process takes a relatively long time as the variable dimension increases. As an efficient global optimization framework, the convex optimization approach has also been widely adopted for the DP syntheses in various array configurations. In 2005, Bucci et al. first proposed a new approach based on convex optimization that can obtain the low SLL DPs of an arbitrary array with a given geometry by solving a convex optimization problem [14]. Two polarization-controlled DP synthesis methods were developed in [15,16], where the polarization and power pattern constraints were jointly converted into convex constraints. These approaches can synthesize the DPs for the circularly polarized linear arrays, the elliptically polarized circular-arc arrays, as well as the broadband polarization-controlled arrays. In 2014, the semidefinite relaxation techniques were used to simultaneously synthesize the SDPs [17], in which part of the excitation coefficients of the SDP were shared. However, an approximate eigenvalue decomposition is required in this approach [17] to recover the excitations, which may lead to performance degradation of the optimized radiation patterns. An iterative convex optimization method was proposed in [18] for the DP synthesis of asymmetric-aperture conformal arrays. In addition, the alternating direction method of multipliers (ADMM) was employed in [19,20] for the synthesis of low SLL SDPs. Nevertheless, the aforementioned methods primarily focus on sidelobe suppression and polarization control of the DPs, and directivity optimization of the array DP has not yet been addressed.

Directivity, as another important performance index of antenna arrays, has also attracted considerable attention. In 2019, Lei et al. formulated the wide-beam array synthesis as a power-gain optimization problem and employed convex optimization to achieve the minimum-gain maximization in [21], and further extended the framework in [22] by incorporating the dynamic range ratio constraints via the iterative convex approximation, enabling practical wide-beam gain optimization with global optimality guarantees. By introducing a convex optimization framework and some mathematical techniques, the nonconvex directivity maximization problems were transformed into the two-stage iterative convex optimization processes in [23,24] to synthesize the high-performance pencil beams and wide beams under the SLL constraints. Furthermore, convex optimization has been extended to the directivity optimization of irregular arrays [25,26,27,28], sparse arrays [29], and multibeam subarray partitioning structures [30,31]. However, these existing directivity maximization methods are mainly devoted to the directivity maximization of pencil-beam, shaped-beam [32], and wide-beam, and specific requirements of the DPs, such as null depth, slope, and balanced directivities of two main lobes, have not yet been addressed. As a result, these methods are not directly applicable to the DP synthesis with directivity maximization.

In this paper, a novel DP synthesis method based on convex optimization is proposed to maximize the DP directivity with the SLL and slope constraints. To the best of the authors’ knowledge, the main contributions of this work with respect to the existing works are highlighted as follows. (1) To maximize the directivity while simultaneously synthesizing the low SLL DP with a large slope, a new nonconvex problem is established. (2) In order to deal with the nonconvexity, some mathematical transformations and the first-order Taylor expansion of the quadratic function are introduced, and the objective function of the directivity maximization is approximated as a linear convex function. By setting the reference phase of the DP slope, the nonconvex constraints are transformed into convex ones. Then, the original nonconvex problem is converted into a series of convex subproblems, which are efficiently solved by the convex optimization solver. In addition, a novel initialization strategy is developed to ensure feasibility and convergence of the proposed iterative scheme. Finally, three numerical examples, including a radome-enclosed linear array, a uniform planar array, and a radome-enclosed planar array, are presented to demonstrate the effectiveness and practicality of the proposed method.

## 2. Problem Formulation

Consider a planar array with L=M×N antenna elements, which are arbitrarily arranged on the *xoy* plane. Let the position coordinates of the *l*-th element be denoted as $\left(x_{l},y_{l}\right)$. The far-field direction of the array is characterized by the azimuth angle φ and the elevation angle θ. The array steering vector is expressed as

(1) $$a(\theta ,\varphi )=\left(g_{1}(\theta ,\varphi )e^{jk(x_{1}u+y_{1}v)},\ldots ,g_{L}(\theta ,\varphi )e^{jk(x_{L}u+y_{L}v)}\right)$$

where k=2π/λ represents the wavenumber, u=sinθcosφ, v=sinθsinφ, and $g_{l}(\theta ,\varphi )$ is the active element pattern (AEP) of the *l*-th element. The AEPs are obtained by exciting one element at a time while terminating all other elements with 50 Ω matched loads, thus the mutual coupling and radome scattering effects are accurately incorporated into the steering vectors used in the optimization.

Given the excitation vector $w={\left(w_{1},w_{2},\ldots ,w_{L}\right)}^{T}$, the array DP is defined as

(2) $$F_{d}(w,\theta ,\varphi )=a(\theta ,\varphi )w$$

The directivity of the planar array in the direction (θ,φ) can be expressed as

(3) $$D(w,\theta ,\varphi )=\frac{4\pi {\left|F_{d}(w,\theta ,\varphi )\right|}^{2}}{\int_{0}^{2\pi }\int_{0}^{\pi }{\left|F_{d}(w,\theta ,\varphi )\right|}^{2}\sin\theta d\theta d\varphi }$$

For mathematical convenience, Equation (3) can be represented in the following matrix form

(4) $$D(w,\theta ,\varphi )=\frac{w^{H}A(\theta ,\varphi )w}{w^{H}Bw}$$

where

(5) $$A(\theta ,\varphi )=a{\left(\theta ,\varphi \right)}^{H}a(\theta ,\varphi )$$

(6) $$B=\frac{1}{4\pi }\int_{0}^{2\pi }\int_{0}^{\pi }a{\left(\theta ,\varphi \right)}^{H}a(\theta ,\varphi )\sin\theta d\theta d\varphi$$

Both matrices ***A*** and ***B*** are Hermitian, and ***B*** is positive definite [24]. For any arbitrary planar array, once the element positions, operating frequency, and target direction (θ,φ) are specified, ***A*** and ***B*** are completely determined.

To ensure the optimization model remains practically accurate, the effects of mutual coupling and radome scattering are rigorously incorporated by using the AEPs. Unlike the ideal array factor model, the steering vector a(θ,φ) in (1) is constructed by using the AEPs, $g_{l}(\theta ,\varphi )$, obtained from the full-wave HFSS simulations. Mathematically, $g_{l}(\theta ,\varphi )$ encompasses the direct radiation of the *l*-th element, the re-radiation from other coupled elements and the scattering/reflections from the radome structure. By embedding these interactions directly into a(θ,φ), the pattern $F_{d}(w,\theta ,\varphi )$ in (2) and the directivity D(w,θ,φ) in (4) account for the radome-induced distortions. Consequently, the matrices A(θ,φ) and B utilized in the optimization subproblems (23) encapsulate all relevant electromagnetic coupling and scattering effects, ensuring that the synthesized weights are optimized for the actual integrated system rather than an idealized model.

In the monopulse radar systems, the DP design requirements are different from those of the sum pattern (SP). For the SP, the directivity in the target direction should be as high as possible, and the peak SLL in the sidelobe region should be as low as possible. However, for the DP, a deep null in the target direction and low SLLs are required at the same time. Moreover, in order to improve angular estimation accuracy, steep slopes on both sides of the DP null are also required. For the problem of maximizing the DP directivity under the SLL constraints, usually the following four design objectives should be achieved.

(1) Null Depth (ND) Constraint: A deep null depth must be formed in the target direction $\left(\theta_{0},\varphi_{0}\right)$, such that
  (7) $$F_{d}(w,\theta_{0},\varphi_{0})=0$$
(2) Slope Constraint: For an elevation DP, the slope in the target direction $\left(\theta_{0},\varphi_{0}\right)$ must satisfy
  (8) $${\left|\frac{\partial F_{d}(w,\theta ,\varphi_{0})}{\partial \theta }\right|}_{\theta =\theta_{0}}\geq S$$

For an azimuth DP, the corresponding slope constraint is

(9) $${\left|\frac{\partial F_{d}(w,\theta_{0},\varphi )}{\partial \varphi }\right|}_{\varphi =\varphi_{0}}\geq S$$

where *S* denotes the prescribed minimum slope. Two slopes of the DP are respectively expressed as

(10) $$\frac{\partial F_{d}(w,\theta ,\varphi )}{\partial \theta }={\tilde{a}}_{el}(\theta ,\varphi )w$$

(11) $$\frac{\partial F_{d}(w,\theta ,\varphi )}{\partial \varphi }={\tilde{a}}_{az}(\theta ,\varphi )w$$

where

(12) $$\begin{array}{l} {\tilde{a}}_{el}(\theta ,\varphi )=(jk(x_{1}\cos\theta \cos\varphi +y_{1}\cos\theta \sin\varphi )g_{1}(\theta ,\varphi )e^{jk(x_{1}u+y_{1}v)},\ldots ,\\ jk(x_{L}\cos\theta \cos\varphi +y_{L}\cos\theta \sin\varphi )g_{L}(\theta ,\varphi )e^{jk(x_{L}u+y_{L}v)}) \end{array}$$

(13) $$\begin{array}{l} {\tilde{a}}_{az}(\theta ,\varphi )=(jk(-x_{1}\sin\theta \sin\varphi +y_{1}\sin\theta \cos\varphi )g_{1}(\theta ,\varphi )e^{jk(x_{1}u+y_{1}v)},\ldots ,\\ jk(x_{L}\sin\theta \sin\varphi +y_{L}\sin\theta \cos\varphi )g_{L}(\theta ,\varphi )e^{jk(x_{L}u+y_{L}v)}) \end{array}$$

(3) SLL Suppression: The peak SLL within the sidelobe region Θ is less than a given upper bound ρ, i.e.,
  (14) $$\left|F_{d}(w,\theta_{q},\varphi_{q})\right|\leq \rho ,(\theta_{q},\varphi_{q})\in \Theta$$
(4) Directivity Maximization: For the DP, usually, two main lobes are symmetrically located on both sides of the null. In order to maximize the directivity of these two main lobes while minimizing their imbalance, the optimization objective is formulated as

(15) $$\min\left\{D(w,\theta_{0}+\Delta \theta ,\varphi_{0}),D(w,\theta_{0}-\Delta \theta ,\varphi_{0})\right\}$$

where Δθ is the angle between the main beam direction and the target direction.

By discretizing the sidelobe region Θ into *Q* angular sampling points $\left\{\left(\theta_{1},\varphi_{1}),(\theta_{2},\varphi_{2}),\ldots ,(\theta_{Q},\varphi_{Q}\right)\right\}$ and incorporating the above design requirements, the azimuth DP directivity maximization problem with the SLL and ND constraints can be formulated as

(16) $$\begin{array}{ll} \underset{w}{\max} & \min\left\{D(w,\theta_{0}+\Delta \theta ,\varphi_{0}),D(w,\theta_{0}-\Delta \theta ,\varphi_{0}))\right\}\\ s.t. & F_{d}(w,\theta_{0},\varphi_{0})=0\\ & {\left|\frac{\partial F_{d}(w,\theta ,\varphi_{0})}{\partial \theta }\right|}_{\theta =\theta_{0}}\geq S\\ & \left|F_{d}(w,\theta_{q},\varphi_{q})\right|\leq \rho ,(\theta_{q},\varphi_{q})\in \Theta ,\;q=1,2,\ldots ,Q \end{array}$$

Similarly, the elevation DP directivity maximization problem is constructed by replacing the azimuth slope constraint in (16) with the elevation slope constraint (9). Problem (16) is nonconvex, since the objective function and the second constraint in Problem (16) are all nonconvex.

## 3. Proposed Method

In this section, a novel method based on the first-order Taylor expansion of a quadratic function is proposed, and the DP directivity maximization problem is equivalently transformed into a series of convex subproblems, which can be optimally solved in polynomial time. Then, an iterative method is designed to obtain solutions of the convex subproblems.

For a real planar array, $w^{H}Bw$ in Equation (4) represents the total power radiated or received by the antenna array over the entire space. For any nonzero excitation vector w, it holds that $w^{H}Bw>0$. Therefore, ***B*** is a Hermitian positive-definite matrix, and there always exists a Hermitian positive-definite matrix ***C*** such that $B=C^{H}C$. Let $\tilde{w}=Cw$, the total radiated (or received) power of the array can be rewritten as $w^{H}Bw={\tilde{w}}^{H}\tilde{w}$, and the directivity in Equation (4) can be equivalently expressed as

(17) $$D(\theta ,\varphi )=\frac{{\tilde{w}}^{H}\tilde{A}(\theta ,\varphi )\tilde{w}}{{\tilde{w}}^{H}\tilde{w}}$$

where $\tilde{A}(\theta ,\varphi )=C^{-H}A(\theta ,\varphi )C^{-1}$.

First, by substituting (7), (10) and (17) into Problem (16) and introducing a slack variable *t*, Problem (16) can be reformulated as

(18) $$\begin{array}{ll} \underset{\tilde{w}\in C^{L},t\in R_{+}}{\max} & t\\ s.t. & {\tilde{w}}^{H}\tilde{A}(\theta_{0}+\Delta \theta ,\varphi_{0})\tilde{w}\geq c_{0}t\\ & {\tilde{w}}^{H}\tilde{A}(\theta_{0}-\Delta \theta ,\varphi_{0})\tilde{w}\geq c_{0}t\\ & {\tilde{w}}^{H}\tilde{w}=c_{0}\\ & a(\theta_{0},\varphi_{0})w=0\\ & \left|{\tilde{a}}_{el}(\theta_{0},\varphi_{0})w\right|\geq S\\ & \left|a(\theta_{q},\varphi_{q})w\right|\leq \rho ,(\theta_{q},\varphi_{q})\in \Theta ,\;q=1,2,\ldots ,Q \end{array}$$

where $c_{0}$ is a constant, and its value will not affect the optimal solution. Since the directivities of the two main lobes are maximized simultaneously and the phase information is unknown, the conventional SP directivity maximization method is no longer applicable. In addition, the equality constraint ${\tilde{w}}^{H}\tilde{w}=c_{0}$ and the DP slope constraint $\left|{\tilde{a}}_{el}(\theta_{0},\varphi_{0})w\right|\geq S$ in Problem (18) are all nonconvex.

Fortunately, the ND and SLL constraints in Problem (18) are convex. To improve the computer efficiency and make the best use of the convexity, the following equivalent transformations are performed, and the nonconvex Problem (18) is then transformed into a standard convex optimization problem.

(1) Since the DP slope is independent of its phase, the DP slope constraint $\left|{\tilde{a}}_{el}(\theta_{0},\varphi_{0})w\right|\geq S$ is equivalent to setting its imaginary part as zero and requiring the real part to be greater than *S*, i.e.,
  (19) Re(a˜el(θ0,φ0)w)≥SIm(a˜el(θ0,φ0)w)=0
(2) The equality constraint ${\tilde{w}}^{H}\tilde{w}=c_{0}$ is relaxed to an inequality constraint ${\tilde{w}}^{H}\tilde{w}\leq c_{0}$. For any solution satisfying the relaxed constraint, one can always obtain another feasible solution by appropriately scaling the excitation vector, which yields a strictly better directivity value.
(3) Two nonconvex constraints ${\tilde{w}}^{H}\tilde{A}(\theta_{0}+\Delta \theta ,\varphi_{0})\tilde{w}\geq c_{0}t$ and ${\tilde{w}}^{H}\tilde{A}(\theta_{0}-\Delta \theta ,\varphi_{0})\tilde{w}\geq c_{0}t$ are handled as follows. The radiated power is defined as a quadratic function
  (20) $$f(\tilde{w})={\tilde{w}}^{H}A(\theta ,\varphi )\tilde{w},\tilde{w}\in C^{L}$$

By using the first-order Taylor expansion, the function (20) is expanded at a point ${\tilde{w}}_{k}$, which is a solution satisfied all the constraints in Problem (20). The linear approximation of the function (20) is given by

(21) f(w˜)≈f(w˜k)+2Re(w˜kHA(θ,φ)(w˜−w˜k))

Let $\delta =\tilde{w}-{\tilde{w}}_{k}$, where $\delta \in C^{L}$ is a perturbation vector. Then, the two nonconvex main lobe radiated power constraints in Problem (18) can be transformed into the following two convex constraints

(22) w˜kHA(θ0+Δθ,φ0)w˜k+2Re(w˜kHA(θ0+Δθ,φ0)δ)≥c0tw˜kHA(θ0−Δθ,φ0)w˜k+2Re(w˜kHA(θ0−Δθ,φ0)δ)≥c0t

In this way, Problem (18) is converted into a series of convex subproblems, and each subproblem can be efficiently solved by using the CVX Tool Box [33]. At the *k*-th iteration, the convex subproblem can be expressed as

(23) minδ∈CL,t∈R+ −ts.t.w˜kHA(θ0+Δθ,φ0)w˜k+2Re(w˜kHA(θ0+Δθ,φ0)δ)≥c0tw˜kHA(θ0−Δθ,φ0)w˜k+2Re(w˜kHA(θ0−Δθ,φ0)δ)≥c0ta(θ0,φ0)(w˜k+δ)=0Re(a˜el(θ0,φ0)(w˜k+δ))≥SIm(a˜el(θ0,φ0)(w˜k+δ))=0a(θq,φq)(w˜k+δ)≤ρ,(θq,φq)∈Θ,q=1,2,…,Q(w˜k+δ)H(w˜k+δ)≤c0δ2≤δmax

where ${\tilde{w}}_{k}$ is the initial solution at the *k*-th iteration, and $\delta_{\max}$ is a step-size parameter to guarantee the accuracy of the linear approximation. By solving Problem (23), δ is obtained, the optimal solution of Problem (18) is updated by ${\tilde{w}}_{k+1}={\tilde{w}}_{k}+\delta$.

It should be noted that each convex subproblem converges only within its sub-domain, and its optimal solution is a local solution of Problem (18). Therefore, a good initial point ${\tilde{w}}_{0}$ is critical, which can improve the probability of converging to the global optimal solution and reduce the number of iterations. However, the constraints in Problem (18) are extremely complicated, and it is difficult to find an initial feasible solution by the analytical method. In this paper, a convex optimization problem is solved to obtain a feasible initial point ${\tilde{w}}_{0}$, which can be formulated as

(24) minw˜0−Ss.t.Re(a˜el(θ0,φ0)w˜0)≥SIm(a˜el(θ0,φ0)w˜0)=0w˜0Hw˜0≤c0a(θ0,φ0)w˜0=0a(θq,φq)w˜0≤ρ,(θq,φq)∈Θ,q=1,2,…,Q

Obviously, in Problem (24), the DP slope is maximized subject to the similar constraints in Problem (18). By solving Problem (24), an initial solution for Problem (23) is obtained. Ultimately, the low SLL DP synthesis problem with maximum directivity is solved iteratively by the convex optimization method.

## 4. Numerical Examples

To verify the effectiveness of the proposed method, three representative examples, namely, a 20-element radome-enclosed linear array, a 16 × 16-element planar array, and a 16 × 16-element radome-enclosed planar array, are synthesized in this section. To accurately evaluate the electromagnetic impact of the radome, the radome-enclosed arrays are simulated by using Ansys HFSS. The entire array structure and the radome are modeled together as an integrated electromagnetic system. For the 20-element radome-enclosed linear array and the 16 × 16-element planar array, a radiation boundary condition is applied to the air box, which is set at a $\lambda_{0}/4$ distance from the outermost structure. For the 16 × 16-element radome-enclosed planar array, due to the excessively large electrical dimensions of the radome, it is difficult to conduct an accurate full-wave simulation solution. Therefore, a FE-BI boundary condition is applied to the air box, which only includes the array antenna structure and is set at a $\lambda_{0}/4$ distance from the planar array structure, and the radome is set to the IE region. The AEPs are extracted by sequentially exciting each port while others are matched, ensuring the mutual coupling and radome effects are included in the optimization model. The AEPs are different from the isolated pattern due to the unavoidable mutual coupling effect between the elements and the radome. To ensure the reproducibility of the results, it is necessary to clarify that the results presented in this section are generated via the analytical superposition using the HFSS-derived AEPs. All the simulations are performed on a computer with an Intel i7-10510U, 1.8 GHz CPU, and 32 GB RAM. The Mosek solver of the CVX toolbox is used during the optimization process [33]. Unless stated otherwise, in our simulations, we choose $c_{0}=1$, $\delta_{\max}=0.02$, S=20, and the sample interval is 0.1°.

### 4.1. The 20-Element Radome-Enclosed Linear Array

**Figure 1:**
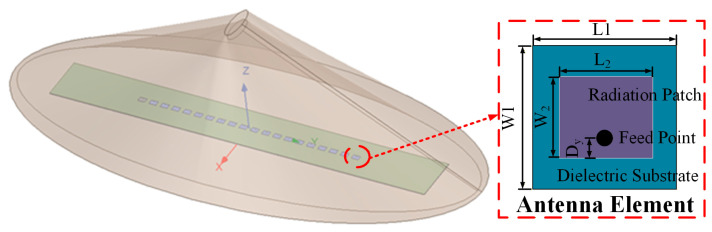
Structures of the 20-element radome-enclosed linear array and the microstrip patch antenna element.

In the first numerical example, a 20-element radome-enclosed linear array is considered. Structures of the linear array are shown in Figure 1. A coax-fed microstrip patch antenna working at 10 GHz, which consists of a radiation patch, a feeding probe, a ground, and a 5 mm thick dielectric layer with relative permittivity 4.4, is chosen as the array element. A probe that connects the radiation patch and the ground is embedded in the substrate layer to feed the antenna. The structural parameters [L1, W1, L2, W2, and Dy] of this antenna element are chosen as [15 mm, 15 mm, 6.45 mm, 9.13 mm, and 1.5 mm]. In order to mimic the real-array environment, an asymmetric flat conical radome with relative permittivity 2.65 is designed, which is placed directly above the linear array to protect the array antenna. The width of the radome is 280 mm, its length is 620 mm, its height is 160 mm, and its thickness is 30 mm.

Firstly, to examine the impact of Δ*θ* on the DP performance, we set Δ*θ* ranging from 5° to 20°, and two main-lobe directivities of the DP are maximized. The target direction $\left(\theta_{0},\varphi_{0}\right)$ is set to (0°, 0°), the desired peak SLL is less than −20 dB, and the desired null depth is less than −50 dB.

**Figure 2:**
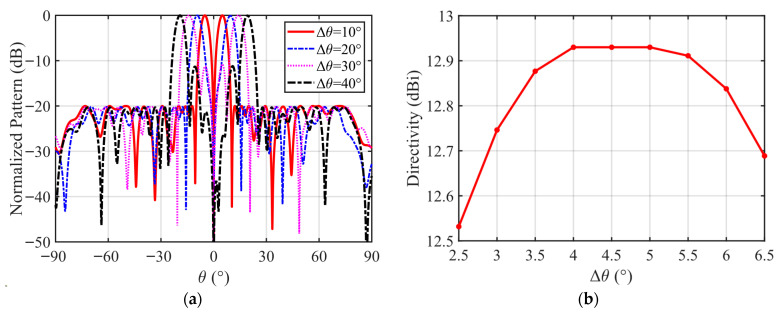
The optimal DPs and directivities for different Δ*θ*: (**a**) the normalized DPs; (**b**) the directivity variation.

The results of the DPs for different Δ*θ*’s are presented in Figure 2, with the normalized DPs depicted in Figure 2a, and the main-lobe directivity variation given in Figure 2b. As shown in this figure, when Δ*θ* is smaller, the DP slope at the boresight is larger but the directivities will decrease. When Δ*θ* is too large, the DP main beam will become two split beams. Figure 2b further illustrates the effect of Δ*θ* on the DP directivities with the same SLL. When Δθ∈[4°,5°], the DP directivities are all greater than 12.9 dBi. Therefore, as long as we set Δ*θ* within this range, the DP directivities will achieve the optimal value. Therefore, in order to enhance universality of the proposed method and avoid the situation where the proposed method fails to converge due to the improper parameter choice for Δ*θ*, an optimal parameter selection strategy is applied in this paper. In this strategy, the initial DP obtained by solving Problem (24) is taken as the target, and the two main-lobe directivities of the DP are maximized.

To test the performance of the proposed method, the low SLL DPs with different scanning angles are synthesized. The optimization objective is to maximize two main-lobe directivities of the DP. The peak SLL is less than −20 dB, and the ND is less than −50 dB. The target directions are respectively set to 0° and 30°. The sidelobe regions are respectively chosen as [−90°,−4.5°]∪[4.5°,90°] and [−90°,22°]∪[38°,90°]. The Bayliss method [3], the convex optimization methods [14,18], ADMM [20], and the proposed method are used to synthesize the low SLL DPs, respectively.

**Figure 3:**
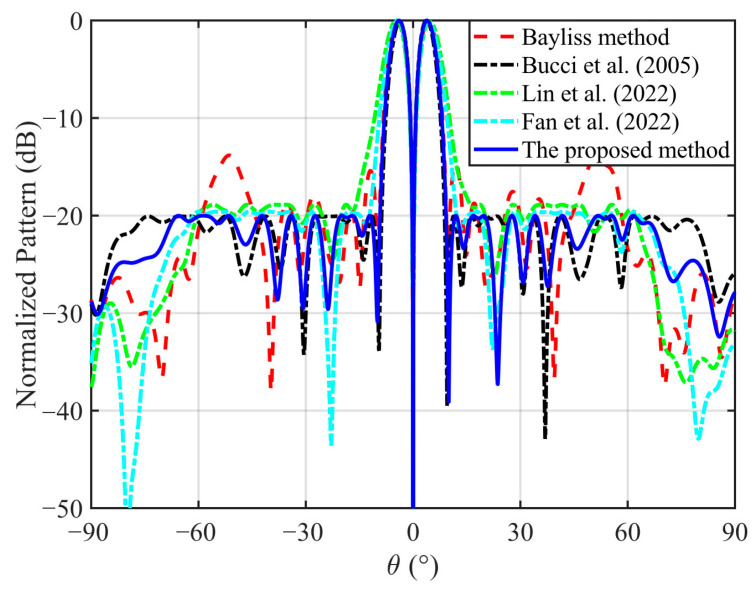
The normalized DPs with the 0° scan angle obtained by five different methods [3,14,18,20].

The normalized DPs with the 0° scan angle obtained by five different methods are compared in Figure 3, and Table 1 lists the relevant data. The proposed method can be observed to not only satisfy the peak SLL constraint but also achieve the deepest ND while attaining the highest directivity. Although the Bayliss method yields a relatively high directivity (12.58 dBi), it fails to satisfy the prescribed SLL requirement due to the radome-induced distortion, with the SLL degrading to −13.82 dB. This demonstrates that classical closed-form distributions cannot compensate for the radome scattering effects. In addition, the optimization-based approaches in [14,18,20] all successfully suppress the SLL and ND. However, their directivities (12.27 dBi, 11.62 dBi, and 11.86 dBi, respectively) are notably lower than the 12.93 dBi achieved by the proposed method. These results indicate that only the SLL and slope constraints are considered in [14,18,20], thus their obtained directivities are lower.

**Figure 4:**
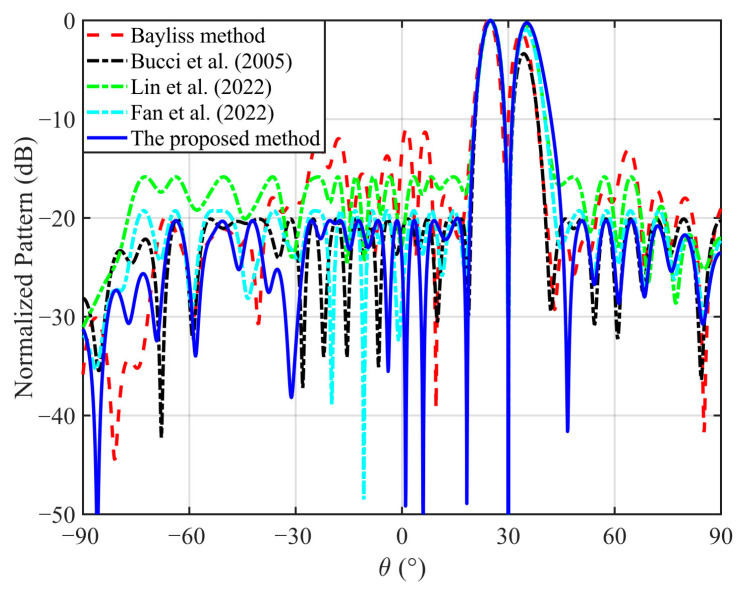
The normalized DPs with the 30° scan angle obtained by five different methods [3,14,18,20].

The normalized DPs with the 30° scan angle obtained by five different methods are plotted in Figure 4, and the specific values are listed in Table 2, where Δ*D* denotes the directivity difference between the two main lobes of the DP. As shown in this figure and table, it can be concluded that the DPs obtained by the method in [14] and the proposed method meet the desired specifications of SLL and ND, but the directivity obtained by the proposed method is 1.67 dB higher than that obtained by the method in [14]. In addition, Δ*D* = 0.25 dB obtained by the proposed method is the smallest, indicating that the two main lobes of the DP are perfectly symmetrical. In contrast, ref. [14] exhibits a much larger imbalance (Δ*D* = 3.37 dB), demonstrating that the slope maximization does not inherently enforce the main-lobe symmetry. The methods in [18,20] improve the symmetry compared to the method in [14], but still do not reach the balance achieved by the proposed formulation. It is worth mentioning that although the directivity obtained by the Bayliss method [3] is the highest, this method [3] fails to compensate for the deteriorating effect of the radome on the array radiation pattern, such as the null correction and the ND optimization, which limits its application. The above results confirm the effectiveness and superiority of the proposed method.

**Figure 5:**
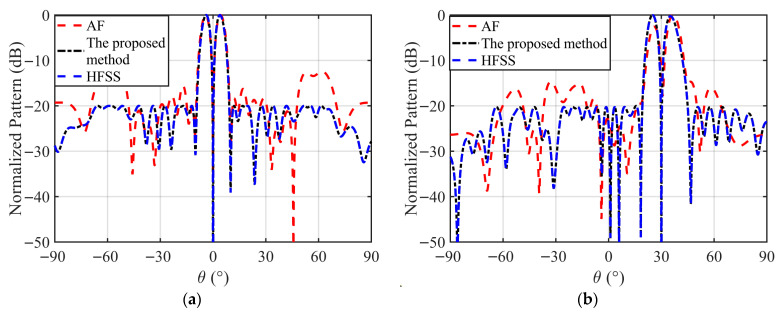
The normalized DPs obtained by the ideal array factor, the proposed method, and the full-wave simulation method. (**a**) the 0° scan angle; (**b**) the 30° scan angle.

The superior directivity performance of the proposed method compared to the traditional convex optimization approach in [14,18] stems from the fundamental difference in optimization objectives. In [14,18], the objective is to maximize the slope of the DP in the null direction, while the SLL and ND are imposed as constraints. Although this formulation ensures a steep null and controlled sidelobes, it does not explicitly optimize the directivities of the two main lobes, and, thus, the achievable directivities are relatively low. In contrast, the proposed method establishes a new optimization model where maximization of the directivities for two symmetric main lobes is the primary objective, while the requirements for the slope, ND, and SLL are all simultaneously satisfied as constraints. This leads to a noticeable increase in the directivities, as well as the improved balance between the two main lobes. The symmetry improvement is particularly evident in Figure 5, where two main lobes synthesized by the proposed approach exhibit nearly identical peak levels, whereas the method in [14] shows observable lobe imbalance. Ultimately, these results demonstrate that the proposed method achieves higher directivities and main-lobe symmetry while satisfying the prescribed slope, ND, and SLL constraints, thereby validating the superiority of the proposed approach in the DP synthesis.

To verify the effectiveness of the proposed method, a full-wave simulation of the 20-element radome-enclosed linear array is performed by using the optimized complex excitations. Table 3 lists the normalized amplitudes and phases for each array element corresponding to the 0° and 30° scan angles. Figure 5 presents the comparison between the optimized patterns and the full-wave simulation results. As shown in this figure, the two patterns obtained by the proposed method and the full-wave simulation method are in good agreement, while the results obtained by the ideal array factor are completely different. The directivities obtained by the full-wave simulation for the 0° and 30° scan angles are 12.90 dBi and 9.98 dBi, respectively, which deviate by only approximately 0.1 dB from the optimization results. The peak SLL and ND from the full-wave simulation at 0° scan angle are −20.00 dB and −74.92 dB, respectively. The SLL and ND at 30° scan angle are −20.03 dB and −80.15 dB, respectively, which achieve the theoretical design objectives. This quantitative agreement confirms that using the HFSS-derived AEPs in our model successfully incorporates the complex mutual coupling and radome scattering effects, validating the reliability of the proposed synthesis technique for real-world applications.

**Figure 6:**
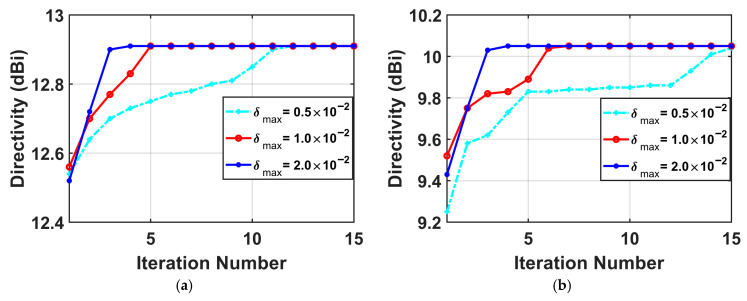
Convergence curves of the proposed method with different $\delta_{\max}$ values. (**a**) the 0° scan angle; (**b**) the 30° scan angle.

Effects of different $\delta_{\max}$ values on convergence of the proposed method are presented in Figure 6. As shown in this figure, the bound value for the linear approximation region mainly influences the convergence speed. When the accuracy of the linear approximation is guaranteed, larger $\delta_{\max}$ will speed up the convergence, while the optimized results are almost identical to those obtained by smaller $\delta_{\max}$ values. Thus, we set $\delta_{\max}=0.02$ for all the numerical examples in this paper.

### 4.2. The 16 × 16-Element Planar Array

**Figure 7:**
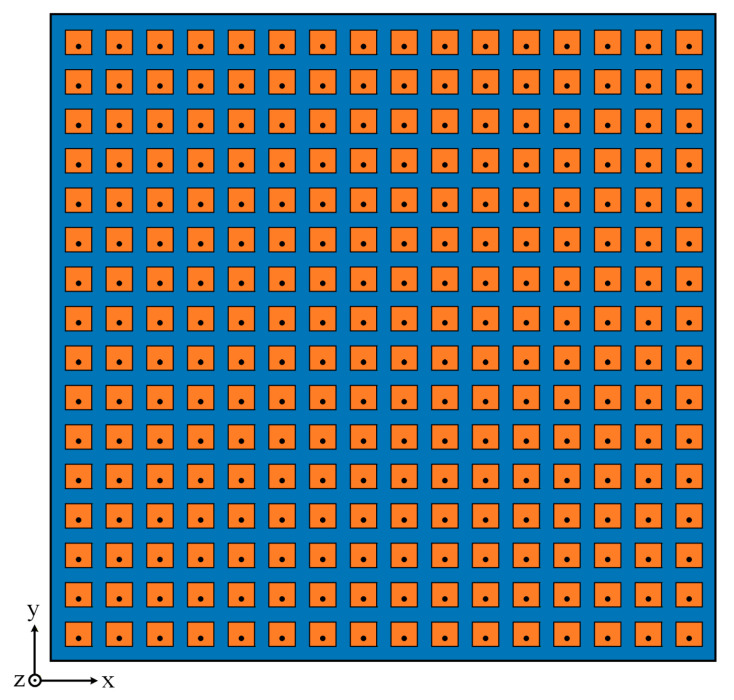
Structure of the 16 × 16-element symmetric planar array.

In the second numerical example, to further verify the ability of the proposed method to synthesize the low SLL and high directivity DP of the planar array, a 16 × 16-element planar array is synthesized, and the array element spacing is 15 mm. The array structure is shown in Figure 7, and the array element structure is shown in Figure 1. The optimization objective is to maximize the two main lobe directivities of the DP. The target direction $\left(\theta_{0},\varphi_{0}\right)$ is set to (0°,45°). The desired peak SLL is less than −30 dB within the sidelobe region $\frac{{\left(u-0.7\right)}^{2}}{{\left(0.52\right)}^{2}}+\frac{v^{2}}{{\left(0.42\right)}^{2}}\geq 1$. The desired null depth is less than −50 dB. The proposed method and the two existing methods proposed in [3,14] are used to solve this DP synthesis problem, respectively.

**Figure 8:**
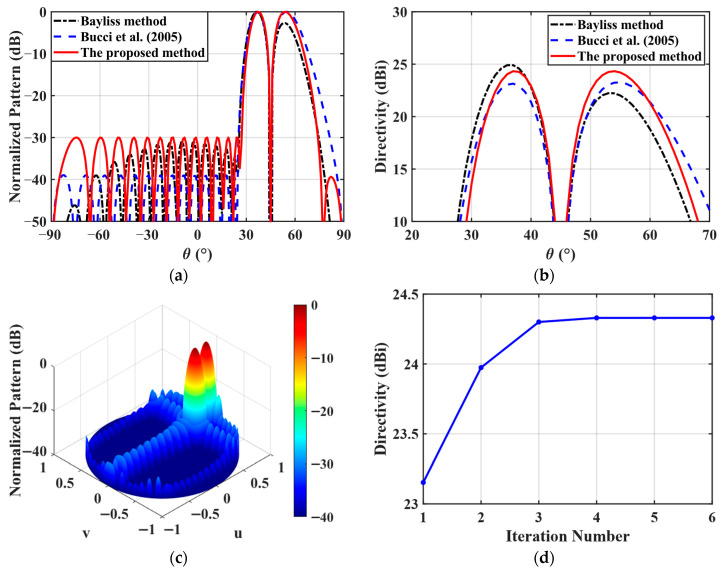
The optimized radiation patterns of the 16 × 16-element planar array. (**a**) The normalized 2D DPs obtained by three different methods [3,14]; (**b**) directivity comparison of the DPs [3,14]; (**c**) the normalized 3D DP; (**d**) Convergence curve of the proposed method.

The optimized DPs obtained by the three methods are illustrated in Figure 8a,b, and the corresponding detailed values, including the directivity, SLL, and ND, are summarized in Table 4. For the azimuth DP synthesis, a −30 dB Bayliss distribution is employed along the *x*-direction to generate the DP, while a −30 dB Taylor distribution is employed along the *y*-direction to form the sum pattern and effectively suppress the SLLs. As reported in Table 3, the proposed method exhibits significantly superior performance compared with the other two existing methods [3,14]. Under the identical SLL and ND constraints, the directivity achieved by the proposed method is 2.11 dB and 1.19 dB higher than those obtained by the Bayliss method [3] and the method in [14], respectively. More importantly, the directivity imbalance between the two main lobes of the DP, i.e., Δ*D*, is not controllable in the other two existing methods [3,14], whereas only the proposed approach is capable of producing a symmetric DP. Figure 8d presents the convergence curve of the proposed method. It follows from this figure that the proposed method converges to the optimal solution within only 4 iterations. This is mainly attributed to the novel initialization strategy for generating a good feasible starting point.

**Figure 9:**
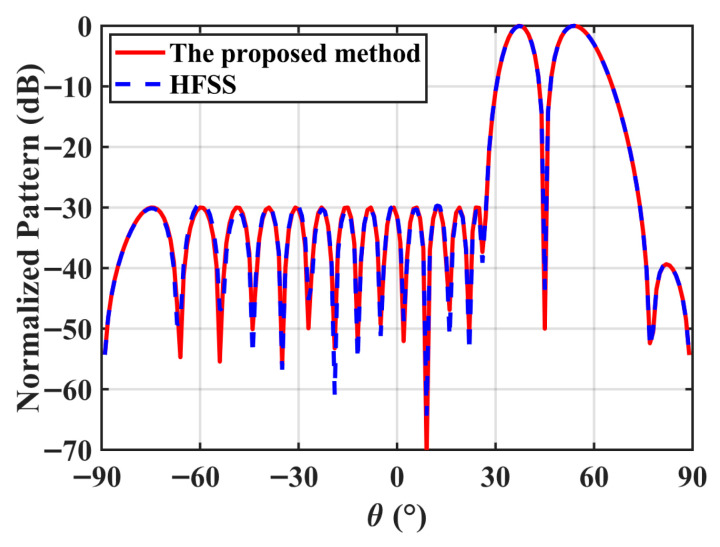
The normalized DPs obtained by the proposed method and the full-wave simulation method.

Figure 9 presents the comparison between the optimized pattern and the full-wave simulation results for the 16 × 16-element symmetric planar array. The full excitation matrix for the 256-element planar array is provided in the Supplementary Material S1. As shown in this figure, the full-wave results closely match the optimized theoretical results. The deviation between theoretical and full-wave directivity values is within 0.1 dB, which confirms that the HFSS-derived AEPs accurately capture the mutual coupling and radome scattering effects. This comparison demonstrates that the proposed convex optimization method is fully consistent with the actual electromagnetic response of the radome-enclosed array system and validates the practical applicability of the synthesized excitation coefficients.

### 4.3. The 16 × 16-Element Radome-Enclosed Planar Array

**Figure 10:**
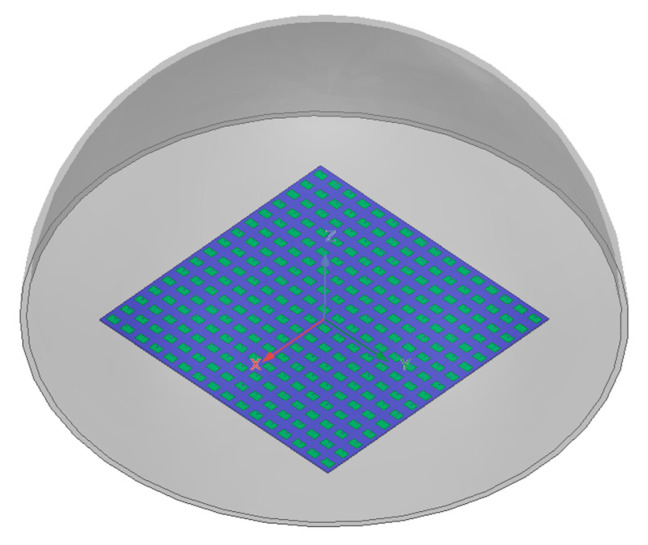
Structure of the 16 × 16-element radome-enclosed planar array.

To further validate the performance of the proposed method under practical conditions, a 16 × 16-element radome-enclosed planar array is simulated and synthesized. A hemispherical radome with a thickness of 5 mm and a relative permittivity of 2.65 is configured above the 16 × 16-element planar array. The radius of the radome is 10 mm. The radome-enclosed planar array structure is shown in Figure 10. The AEPs for the 16 × 16-element radome-enclosed planar array are extracted via the full-wave HFSS simulations.

**Figure 11:**
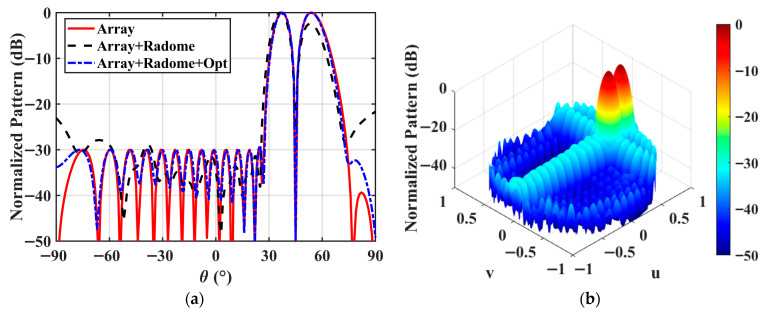
The DPs of the 16 × 16-element planar arrays without and with the radome. (**a**) The normalized 2D DPs; (**b**) the normalized 3D DP.

The DPs of the 16 × 16-element planar arrays without and with the radome are compared in Figure 11. The 16 × 16-element radome-enclosed planar array is excited by using the same excitation coefficients as in Example 2. Parameter comparisons of the DPs of the arrays without and with the radome are given in Table 5. It can be observed that due to the coupling effect of the radome, directivity of the 16 × 16-element radome-enclosed planar array is 23.04 dBi, which decreased by 1.29 dB. The SLL and the ND are −21.65 dB and −25.79 dB, respectively, which increased by 8.35 dB and 24.21 dB. Furthermore, the directivity difference between the two main lobes of the DP is 2.42 dB.

To achieve the −30 dB SLL and −50 dB ND, the proposed directivity maximization method is applied to synthesize the DP for the 16 × 16-element radome-enclosed planar array. The optimized DPs are illustrated in Figure 11a, and the corresponding detailed values are listed in Table 5. The full excitation matrix for the 256-element radome-enclosed planar array is provided in the Supplementary Material S2. The optimized excitation coefficients are subsequently assigned to the complete planar array model for the full-wave validation. As shown in Figure 11 and Table 5, the proposed method successfully compensates for the radome-induced deteriorations, achieving a maximized directivity of 23.82 dBi while satisfying the −30 dB SLL and −50 dB ND constraints. The directivity degradation caused by the radome is limited to approximately 0.51 dB after optimization. This confirms the performance of the proposed design method for large-scale planar arrays in realistic electromagnetic environments.

## 5. Conclusions

In this paper, a novel design method is proposed to maximize the DP directivities for monopulse linear and planar phased arrays. First, a new nonconvex problem for maximizing two main-lobe directivities of the DP while simultaneously synthesizing the low sidelobe DP is established. Then, to address the nonconvexity of the directivity maximization objective, a sequential convex optimization approach based on the first-order Taylor expansion of the quadratic function is developed. By fixing the reference phase of the DP slope, the nonconvex constraints are equivalently converted into the convex ones. Consequently, the original nonconvex problem is decomposed into a series of convex subproblems, which are efficiently solved by standard convex optimization solvers. In addition, an effective initialization strategy is proposed to generate a good, feasible starting point that satisfies the complicated constraints. Finally, three representative examples have been synthesized to demonstrate the excellent performance of the proposed design method.

## Figures and Tables

**Table 1:** Parameter comparison of the optimal DPs with the 0° scan angle.

Algorithm	Directivity (dBi)	SLL (dB)	ND (dB)
Bayliss method [3]	12.58	−13.82	−37.53
Bucci et al. [14]	12.27	−20.00	−50.00
Lin et al. [18]	11.62	−18.88	−50.43
Fan et al. [20]	11.86	−19.61	−50.53
**The proposed method**	**12.93**	**−20.00**	**−55.00**

**Table 2:** Parameter comparison of the optimal DPs with the 30° scan angle.

Algorithm	Directivity (dBi)	Δ*D* (dB)	SLL (dB)	ND (dB)	Null Angle (°)
Bayliss method [3]	10.36	1.24	−10.98	−15.41	29.1
Bucci et al. [14]	8.38	3.37	−20.00	−217.6	30.0
Lin et al. [18]	9.25	0.48	−15.84	−209.81	30.0
Fan et al. [20]	9.40	0.94	19.26	−191.42	30.0
**The proposed method**	**10.05**	**0.25**	**−20.0** **0**	**−** **218.2**	**30.0**

**Table 3:** Amplitudes and phases for each array element corresponding to the 0° and 30° scan angles.

Element Number	Amplitude (V)		Phase (°)		Element Number	Amplitude (V)		Phase (°)	
1	0.84	0.21	268.22	88.70	11	0.39	0.30	31.31	153.51
2	0.60	0.31	232.63	351.42	12	0.43	0.07	112.38	114.02
3	0.96	0.33	235.50	243.81	13	0.78	0.26	60.50	265.62
4	0.63	0.60	256.57	130.76	14	0.76	0.39	64.67	127.04
5	0.91	0.57	237.10	47.53	15	0.89	0.45	82.17	24.09
6	0.93	0.64	266.39	321.45	16	0.91	0.91	57.32	303.84
7	0.76	0.61	247.92	218.27	17	0.64	0.66	70.95	192.46
8	0.85	0.65	240.69	135.71	18	1.00	1.00	57.49	87.53
9	0.43	0.67	292.95	28.25	19	0.67	0.36	53.25	48.97
10	0.39	0.59	211.17	290.18	20	0.83	0.51	87.06	246.12

**Table 4:** Parameter comparison of the optimal DPs with the (0°, 45°) scan angle.

Algorithm	Directivity (dBi)	Δ*D* (dB)	SLL (dB)	Null Depth (dB)
Bayliss method [3]	22.22	2.67	−30.38	−50.0
Bucci et al. [14]	23.14	0.12	−30.00	−50.0
**The proposed method**	**24.33**	**0.00**	**−30.0** **0**	**−50.0**

**Table 5:** Parameter comparisons of the DPs of the 16 × 16-element planar arrays without and with the radome.

	Directivity (dBi)	Δ*D* (dB)	SLL (dB)	Null Depth (dB)
Array	24.33	0.00	−30.00	−50.0
Array + Radome	23.04	2.42	−21.65	−25.79
Array + Radome + Opt	**23.82**	**0.00**	**−** **30.00**	**−** **50.00**

## Data Availability

All data generated or analyzed during this study are included in this manuscript. There are no additional data or datasets beyond what is presented in the manuscript.

## Supplementary Materials

The following supporting information can be downloaded at: https://www.mdpi.com/article/10.3390/mi17030321/s1, Supplementary Material S1. Supplementary Material S2.

## References

[1] Skolnik M.I. (2008). Radar Handbook.

[2] Sherman S.M., Barton D.K. (2011). Monopulse Principles and Techniques.

[3] Bayliss E.T. (1968). Design of monopulse antenna difference patterns with low sidelobes. Bell Syst. Tech. J..

[4] Elliott R. (1976). Design of line source antennas for difference patterns with sidelobes of individually arbitrary heights. IEEE Trans. Antennas Propag..

[5] McNamara D.A. (1993). Direct synthesis of optimum difference patterns for discrete linear arrays using Zolotarev distributions. IEE Proc. H.

[6] McNamara D.A. (1994). Performance of Zolotarev and modified-Zolotarev difference pattern array distributions. IEE Proc. Microw. Antennas Propag..

[7] Oraizi H., Fallahpour M. (2011). Sum, Difference and Shaped Beam Pattern Synthesis by Non-Uniform Spacing and Phase Control. IEEE Trans. Antennas Propag..

[8] Zeng Q., Yang P., Lu X., Luo M., Man Y., Yang F., Yang S. (2022). Synthesis of Simultaneous Sum and Difference Patterns in Single-Channel 1-Bit Time-Modulated Array. IEEE Antennas Wireless Propag. Lett..

[9] Caorsi S., Massa A., Pastorino M., Randazzo A. (2005). Optimization of the difference patterns for monopulse antennas by a hybrid real/integer-coded differential evolution method. IEEE Trans. Antennas Propag..

[10] Chen Y., Yang S., Nie Z. (2008). The application of a modified differential evolution strategy to some array pattern synthesis problems. IEEE Trans. Antennas Propag..

[11] Li M., Liu Y., Guo Y.J. (2021). Design of Sum and Difference Patterns by Optimizing Element Rotations and Positions for Linear Dipole Array. IEEE Trans. Antennas Propag..

[12] Yang S.-H., Kiang J.-F. (2014). Optimization of Asymmetrical Difference Pattern with Memetic Algorithm. IEEE Trans. Antennas Propag..

[13] Cui C.Y., Jiao Y.-C., Zhang L., Lu L., Zhang H. (2017). Synthesis of Subarrayed Monopulse Arrays With Contiguous Elements Using a DE Algorithm. IEEE Trans. Antennas Propag..

[14] Bucci O.M., D’Urso M., Isernia T. (2005). Optimal synthesis of difference patterns subject to arbitrary sidelobe bounds by using arbitrary array antennas. IEE Proc. Microw. Antennas Propag..

[15] Li M., Wang X., Dong J., Li Y. (2012). Optimal Difference Pattern Synthesis With Polarization Control for Arbitrary Arrays. IEEE Antennas Wireless Propag. Lett..

[16] Li M., Chang Y., Li Y., Dong J., Wang X. (2013). Optimal polarized pattern synthesis of wideband arrays via convex optimization. IET Micro. Antennas Propag..

[17] Fuchs B. (2014). Application of Convex Relaxation to Array Synthesis Problems. IEEE Trans. Antennas Propag..

[18] Lin H.S., Cheng Y.J., Fan Y. (2022). Synthesis of Difference Patterns for 3-D Conformal Beam-Scanning Arrays With Asymmetric Radiation Aperture. IEEE Trans. Antennas Propag..

[19] Fan X., Liang J., Zhao X., Zhang Y., So H.C. (2020). Optimal Synthesis of Sum and Difference Beam Patterns With a Common Weight Vector for Symmetric and Asymmetric Antenna Arrays. IEEE Trans. Antennas Propag..

[20] Fan X., Liang J., Jing Y., So H.C., Geng Q., Zhao X. (2022). Sum/Difference Pattern Synthesis with Dynamic Range Ratio Control for Arbitrary Arrays. IEEE Trans. Antennas Propag..

[21] Lei S., Yang Y., Hu H., Zhao Z., Chen B., Qiu X. (2019). Power Gain Optimization Method for Wide-beam Array Antenna via Convex Optimization. IEEE Trans. Antennas Propag..

[22] Lei S., Hu H., Tang P., Chen B., Tian J., Yang W., Qiu X. (2019). Power-Gain Pattern Synthesis of Array Antenna with Dynamic Range Ratio Restriction. IEEE Antennas Wireless Propag. Lett..

[23] Zhang Y.-X., Jiao Y.-C., Zhang L. (2020). Efficient Directivity Maximization of Time-Modulated Arrays With Two-Stage Convex Optimization. IEEE Antennas Wireless Propag. Lett..

[24] Zhang Y.-X., Jiao Y.-C., Zhang L. (2021). Antenna Array Directivity Maximization With Sidelobe Level Constraints Using Convex Optimization. IEEE Trans. Antennas Propag..

[25] Ma Y., Yang S., Chen Y., Qu S.-W., Hu J. (2021). High-Directivity Optimization Technique for Irregular Arrays Combined with Maximum Entropy Model. IEEE Trans. Antennas Propag..

[26] Pu S., Dong W., Xu Z., Zeng H., Yang G. (2024). Joint Optimization of Domino Subarray Tiling and Generalized Directivity Based on Iterative Convex Relaxation. IEEE Antennas Wireless Propag. Lett..

[27] Yang F., Ma Y., Long W., Sun L., Chen Y., Qu S.-W. (2021). Synthesis of Irregular Phased Arrays Subject to Constraint on Directivity via Convex Optimization. IEEE Trans. Antennas Propag..

[28] Yang F., Ma Y., Chen Y., Qu S., Yang S. (2022). A Novel Method for Maximum Directivity Synthesis of Irregular Phased Arrays. IEEE Trans. Antennas Propag..

[29] Yang F., Yang S., Chen Y., Qu S., Hu J. (2021). Synthesis of Sparse Antenna Arrays Subject to Constraint on Directivity via Iterative Convex Optimization. IEEE Antennas Wireless Propag. Lett..

[30] Gong Y., Xiao S., Zheng Y., Wang B. (2022). An Effective Hybrid Synthesis Strategy of Multibeam Subarray. IEEE Trans. Antennas Propag..

[31] Zeng H., Xu Z.-H., Yang G.-Q., Dong W., Wang S.-L., Xiao S.-P. (2024). Multibeam Directivity Maximization for Overlapped Subarrayed Array via Alternate Convex Programming. IEEE Antennas Wireless Propag. Lett..

[32] Lei S., Chen B., Lin Z., Yang W., Tian J., Hu H. (2023). Sidelobe-Level Minimization With Power Gain Constraint via a Wide-Beam Antenna Array. IEEE Antennas Wireless Propag. Lett..

[33] CVX Research, Inc. CVX: MATLAB Software for Disciplined Convex Programming, Version 2.2, January 2020. http://cvxr.com/cvx.

